# Methanol-Free Production of Recombinant AppA Phytase in *Komagataella phaffii*: Evaluation of Cultivation Conditions, Bioreactor Cultivation, and Biochemical Characterization

**DOI:** 10.3390/biology15191758

**Published:** 2026-10-02

**Authors:** Magzhan Astrakhanov, Viktoriya Popova, Kairat Baltin, Saveliy Kirillov, Bekbolat Khassenov, Saniya Aktayeva

**Affiliations:** Laboratory for Genetics and Biochemistry of Microorganisms, National Center for Biotechnology, Astana 010000, Kazakhstan

**Keywords:** *Komagataella phaffii*, AppA phytase, constitutive expression, recombinant protein production, N-glycosylation, bioprocess development, stirred-tank bioreactor, protein secretion

## Abstract

Phytases are widely used as feed additives because they improve phosphorus utilization from plant-derived feed ingredients and reduce phosphorus excretion into the environment. Most recombinant AppA phytases produced in *Komagataella phaffii* have been obtained using methanol-inducible expression systems, whereas information on constitutive methanol-free production remains limited. In this study, *Escherichia coli* AppA phytase was expressed in *K. phaffii* under the control of the glyceraldehyde-3-phosphate dehydrogenase promoter. Cultivation temperature and initial glucose concentration were evaluated in shake-flask cultures, while the selected conditions were assessed in a 2 L stirred-tank bioreactor. Extracellular phytase activity reached 114.17 ± 2.71 U/mL in shake flasks at 27 °C with 3% initial glucose. The *K. phaffii*-produced enzyme was N-glycosylated, with an apparent molecular weight of approximately 54 kDa compared with approximately 45 kDa after deglycosylation. Compared with purified rAppA-E, the rAppA-P preparation showed a lower temperature optimum, a narrower pH–activity profile, and lower apparent thermostability. In a single bioreactor run, extracellular phytase activity reached 186.81 ± 0.25 U/mL, approximately 1.6-fold higher than the maximum mean shake-flask value. These findings demonstrate constitutive, methanol-free extracellular production of AppA in *K. phaffii* and support further development of controlled cultivation strategies.

## Simple Summary

Phytase is an enzyme widely used in animal feed because it helps animals utilize phosphorus naturally present in plant materials. This improves feed nutritional value, reduces the need for mineral phosphorus supplements, and can decrease phosphorus release into the environment. Many recombinant phytases are produced using microbial expression systems, but some processes rely on methanol, which complicates fermentation and requires additional safety measures. In this study, we investigated a methanol-free approach for producing bacterial AppA phytase in the yeast *Komagataella phaffii*. We evaluated how cultivation temperature and initial glucose concentration affected enzyme production and then tested the selected cultivation condition in a laboratory stirred-tank bioreactor. The yeast-produced phytase was secreted into the culture medium and was glycosylated. Its biochemical properties differed from those of the corresponding phytase produced in *Escherichia coli*. The results show that constitutive expression in *K. phaffii* supports methanol-free extracellular production of AppA phytase and provides a basis for further improvement of the cultivation process and enzyme properties.

## 1. Introduction

Feed enzymes are widely used in modern animal production to improve the utilization of plant-based feeds, particularly in monogastric animals. Phytases catalyze the stepwise hydrolysis of phytate to inorganic phosphate and intermediate phosphorylated myo-inositol derivatives. Because endogenous phytase activity in monogastric animals is generally insufficient for efficient phytate hydrolysis, much of the phytate-bound phosphorus present in plant-derived feeds remains unavailable. It is mostly excreted, contributing to environmental phosphorus loading and eutrophication [1,2,3,4].

In addition to limiting phosphorus bioavailability, phytate has antinutritional effects through interactions with proteins and other nutrients, which can impair nutrient utilization [5,6]. Dietary phytase supplementation increases phosphorus availability and reduces the antinutritional effects of phytate, making phytases important additives in animal nutrition [7]. Phytases have been identified in bacteria, fungi, plants, and animals [8,9,10,11,12,13,14,15]. Among them, bacterial phytases, particularly *Escherichia coli* AppA, are of considerable interest because of their high catalytic efficiency [16].

Despite the availability of commercial phytase preparations, further improvements in enzyme activity, thermal stability, and production efficiency remain desirable [17,18]. Recombinant production is an important strategy for achieving high enzyme yields because native producers may not provide titres suitable for industrial applications [19]. Expression systems can further enhance recombinant protein production through approaches such as using strong promoters, codon optimization, and increased gene copy number [4].

Yeast expression systems have become attractive alternatives for recombinant phytase production because they combine efficient protein secretion with the ability to perform eukaryotic post-translational modifications [19,20,21,22,23]. Among them, *Komagataella phaffii* (formerly *Pichia pastoris*) has emerged as one of the most widely used production hosts owing to its capacity for high-cell-density cultivation, efficient secretion of heterologous proteins, and established tools for recombinant protein production, including the production and characterization of industrially relevant recombinant enzymes such as milk-clotting proteases [24,25,26,27]. The methanol-inducible alcohol oxidase 1 promoter (AOX1) is one of the most widely used promoters for heterologous protein production in *K. phaffii* [24,25]. However, methanol induction complicates large-scale production because of safety concerns about methanol and the need for careful process control [24,25]. Consequently, constitutive expression driven by the glyceraldehyde-3-phosphate dehydrogenase (GAP) promoter has attracted increasing attention as an alternative strategy for recombinant protein production [21,24,25]. Constitutive expression also simplifies process operation by eliminating the induction phase and enabling synthesis of the target protein throughout cultivation [21,25].

Previous studies have demonstrated heterologous expression of *E. coli* AppA phytase in *K. phaffii*, predominantly using methanol-inducible systems [28,29,30]. They explored various strategies to improve AppA production, such as optimizing cultivation conditions and engineering expression, protein folding, and secretion [21,30]. Navone et al. demonstrated that AppA can be expressed using different regulatory strategies, including constitutive expression under the GAP promoter; however, production with this promoter was relatively low under the conditions tested [21]. Tai et al. reported constitutive AppA production under GAP promoter control [31]. Nevertheless, information on how cultivation parameters, particularly temperature and initial glucose concentration, influence biomass formation, substrate utilization, and extracellular AppA production under constitutive GAP control remains limited [21,31]. Furthermore, although AppA production has been investigated under controlled bioreactor conditions using derepressed and methanol-induced strategies [32], constitutive, methanol-free GAP-driven AppA production under controlled bioreactor conditions remains comparatively underexplored.

The expression host may also influence protein folding and post-translational modifications, including glycosylation, which could affect recombinant protein properties [24,25]. Comparative characterization of AppA preparations produced in bacterial and yeast expression systems may therefore provide useful information on host-associated differences; however, such comparisons require caution when the preparations differ in purification state. The objective of the present study was therefore to evaluate recombinant AppA phytase production in *K. phaffii* under constitutive GAP control and to characterize the biochemical properties of the secreted enzyme compared with its recombinant counterpart produced in *E. coli*. Specifically, we evaluated the effects of cultivation temperature and initial glucose concentration on biomass accumulation, glucose utilization, and extracellular phytase production; evaluated the selected cultivation condition in a 2 L stirred-tank bioreactor; and compared the biochemical properties of rAppA-P and rAppA-E. The results demonstrate efficient constitutive, methanol-free extracellular production of AppA in *K. phaffii*, identify cultivation conditions that favour extracellular phytase production, and support further development of controlled cultivation strategies for this expression system.

## 2. Materials and Methods

### 2.1. Chemical Reagents and Media Components

The principal chemical reagents and media components used in this study were obtained from the following suppliers: imidazole, Coomassie Brilliant Blue R-250, sodium acetate, FeSO_4_, and bromophenol blue from AppliChem (Darmstadt, Germany); glucose, sodium phytate, and SDS from Sigma-Aldrich (St. Louis, MO, USA); kanamycin from SANTO (Shymkent, Kazakhstan); Zeocin™ from Invitrogen (Villebon-sur-Yvette, France); yeast extract and glycerol from TM Media (Delhi, India); peptone from Fluka Analytical (Buchs, Switzerland); Tris from AMRESCO (Solon, OH, USA); NaCl from VWR Chemicals (Leuven, Belgium); Tween 20 from Fisher BioReagents (Fair Lawn, NJ, USA); ammonium molybdate from Laborpharma (Almaty, Kazakhstan); TCA from Reakhim; and KH_2_PO_4_ from Thermo Scientific (Geel, Belgium). Other chemicals were of analytical grade.

### 2.2. Construction of the Bacterial AppA Expression Plasmid

To obtain recombinant AppA phytases for comparative characterization, bacterial and yeast expression constructs were generated. For bacterial expression, the *appA* gene (1296 bp) from *E. coli* BL21(DE3) (GenBank accession no. CP001509; RefSeq NC_012971), including its native signal peptide sequence, was amplified from genomic DNA using the primers AppA-Efw and AppA-Erv (Table S1). Genomic DNA was isolated using the Monarch Nucleic Acid Purification Kit (New England Biolabs, Ipswich, MA, USA).

PCR amplification was performed using Q5 High-Fidelity DNA Polymerase (New England Biolabs, Ipswich, MA, USA) with an initial denaturation at 98 °C for 30 s, followed by 30 cycles of 98 °C for 10 s, 55 °C for 20 s, and 72 °C for 30 s, and a final extension at 72 °C for 2 min.

The amplified *appA* gene and pET-28c (+) vector were digested with *Nco*I and *Not*I and ligated using T4 DNA ligase (Thermo Fisher Scientific, Waltham, MA, USA). *E. coli* DH5α transformants were selected on LB agar containing 50 µg/mL kanamycin and screened via PCR using T7fw and T7rv (Table S1). Positive clones were verified using Sanger sequencing [33]. The resulting pET-28/AppA construct encoded recombinant AppA carrying a C-terminal 6 × His tag.

To construct the bacterial expression strain, competent *E. coli* ArcticExpress (DE3)RP cells were transformed with pET-28/AppA by electroporation using a MicroPulser™ electroporator (Bio-Rad Laboratories, Hercules, CA, USA), a 0.2 cm cuvette, and the EC2 programme at 2.5 kV. Transformants were selected on LB agar containing 50 µg/mL kanamycin. The resulting recombinant *E. coli* ArcticExpress (DE3)RP/pET-28/AppA strain was stored at −80 °C in LB medium containing 50% glycerol.

### 2.3. Construction of the Yeast AppA Expression System

For yeast expression, a codon-optimized *appA* gene was commercially synthesized (Macrogen, Republic of Korea) and supplied in the pTOP_Blunt_V2 vector. Gene amplification was performed using the primers AppA-EcoRI and AppA-NotI (Table S1), which included *Eco*RI and *Not*I restriction sites, respectively. PCR amplification was performed using Q5 High-Fidelity DNA Polymerase (New England Biolabs, Ipswich, MA, USA) under the conditions described above. The amplified *appA* gene and pGAPZαA vector (Invitrogen, Carlsbad, CA, USA) were digested with *Eco*RI and *Not*I and ligated using T4 DNA ligase, placing *appA* downstream of the constitutive GAP promoter and the *Saccharomyces cerevisiae* α-mating factor secretion signal. Following *E. coli* DH5α transformation, transformants were selected on LB agar containing 100 µg/mL zeocin and screened via PCR using primers GAPfw and AOX1rv (Table S1). Positive clones were verified by sequencing, and the resulting plasmid was designated pGAPZαA-*appA*.

To construct the yeast expression strain, the pGAPZαA-*appA* plasmid was linearized with *Bsp*HI (Thermo Fisher Scientific, Waltham, MA, USA) and introduced into competent *K. phaffii* X-33 cells by electroporation according to the EasySelect™ *Pichia* Expression Kit protocol (Invitrogen, USA). Electroporation was performed with 3 µg of linearized plasmid DNA and 80 µL of competent cells in a 0.2 cm cuvette at 2 kV using a MicroPulser™ electroporator (Bio-Rad Laboratories, Hercules, CA, USA), program Pic. Transformants were selected on YPDS (yeast extract–peptone–dextrose–sorbitol) agar containing 200 μg/mL zeocin and screened via PCR using primers GAPfw and AOX1rv (Table S1). This was performed to verify the presence of the *appA* expression cassette. PCR-positive clones were subsequently screened for phytase activity; the clone exhibiting the highest activity was selected as the production strain.

The stability of *appA* integration into the yeast genome was evaluated as described previously [34]. Briefly, the selected strain was cultivated in YPD medium without antibiotic selection for 20 generations, followed by YPD agar cultivation with zeocin and PCR verification of the integrated expression cassette. The recombinant *K. phaffii* X-33 strain harbouring the integrated *appA* expression cassette was stored at −80 °C in YPD medium containing 20% glycerol.

### 2.4. In Silico Analysis of Recombinant AppA Constructs

The cloning strategy for the *appA* gene, including restriction site selection, primer design, in silico analysis of the recombinant constructs, and calculation of theoretical molecular weights and isoelectric points, was developed using Clone Manager 7, version 7.11 (Scientific & Educational Software, Denver, CO, USA). Potential N-linked glycosylation sites in the AppA amino acid sequence [35] and the N-terminal signal peptide and its cleavage site were predicted using NetNGlyc 1.0 and SignalP 5.0, respectively [36]. The amino acid sequences of the recombinant AppA variants produced in *E. coli* and *K. phaffii* were aligned using Clustal Omega version 1.2.4 (EMBL-EBI, Hinxton, UK) [37], and the resulting alignment was visualized using ESPript 3.2 (IBCP, Lyon, France) [38].

### 2.5. Production and Preparation of Recombinant AppA Phytases

For rAppA-E production, individual colonies of the recombinant *E. coli* ArcticExpress (DE3)RP/pET-28/AppA strain were inoculated into LB broth containing 50 µg/mL kanamycin and cultivated at 37 °C until 0.6 OD_600_ was reached. Recombinant protein expression was induced by adding isopropyl β-D-1-thiogalactopyranoside (IPTG) to a final concentration of 0.05 mM, followed by cultivation at 18 °C for 16 h.

Cells were harvested by centrifugation at 6000× *g* for 7 min and resuspended in a 25 mM HEPES-NaOH buffer (pH 7.5) containing 20 mM NaCl. Lysozyme was added to a final concentration of 2 mg/mL, and the cell suspension was incubated at room temperature for 20 min. Cells were subsequently disrupted by sonication for a total of 8 min using alternating 10 s sonication and 20 s pause cycles at 30% amplitude. The resulting lysate was clarified by centrifugation at 40,000× *g* for 1 h at 4 °C, and the supernatant containing the soluble protein fraction was filtered through a 0.22 μm membrane prior to chromatographic purification.

rAppA-E was purified by immobilized metal affinity chromatography using a 1 mL HisTrap crude FF column (Cytiva, Marlborough, MA, USA) on an ÄKTA Purifier 10 system (GE Healthcare, Uppsala, Sweden). The column was equilibrated with 20 mM HEPES-NaOH (pH 7.5), 500 mM NaCl, and 20 mM imidazole, and the protein was eluted with a linear 20–500 mM imidazole gradient at a 0.5 mL/min flow rate. Fractions containing phytase activity were identified using SDS-PAGE, and activity assay results were pooled, concentrated, and buffer-exchanged using 10 kDa MWCO centrifugal concentrators (Pierce™, Thermo Fisher Scientific, Rockford, IL, USA). Purified rAppA-E was stored at 4 °C until further analysis.

rAppA-P was produced using the selected recombinant *K. phaffii* X-33 strain described above. Following cultivation, cells were removed by centrifugation at 10,000× *g* for 10 min at 4 °C, and the supernatant was filtered through a 0.22 µm membrane. The resulting cell-free culture supernatant was used without further purification for phytase activity assays, and pH– and temperature–activity profiles were determined. For SDS-PAGE, Western blotting, and Endo H deglycosylation, the cell-free culture supernatant was concentrated 10-fold by centrifugal ultrafiltration using 10 kDa molecular-weight-cutoff (MWCO) centrifugal filter units (Vivaspin^®^ 2, Sartorius Stedim Biotech, Göttingen, Germany).

### 2.6. SDS-PAGE, Western Blotting, and Deglycosylation

Purified rAppA-E and 10-fold concentrated rAppA-P samples were mixed with a 6× Laemmli sample buffer, heated at 95 °C for 5 min, and separated on 12% SDS–polyacrylamide gels. Protein bands were visualized by staining with 0.1% (*w*/*v*) Coomassie Brilliant Blue R-250.

For Western blotting, proteins were transferred onto polyvinylidene difluoride (PVDF) membranes (Pierce™, Thermo Scientific, Rockford, IL, USA). Membranes were blocked, and antibodies were incubated using 5% (*w*/*v*) non-fat dry milk in TBST (50 mM Tris-HCl, pH 7.6, 150 mM NaCl, and 0.1% [*v*/*v*] Tween 20).

Animal maintenance and experimental procedures were approved by the Local Ethics Committee of the National Center for Biotechnology (Astana, Kazakhstan) and were conducted in accordance with the national regulations of Kazakhstan and the European Convention for the Protection of Vertebrate Animals Used for Experimental and Other Scientific Purposes (ETS No. 123, Strasbourg, 1986). Rabbit polyclonal antibodies against recombinant AppA were generated in-house. A rabbit was immunized six times with purified recombinant AppA (0.15–0.30 mg per immunization) over a 35-day period. Following hyperimmune serum collection, immunoglobulins were enriched by fractional ammonium sulfate precipitation. Ammonium sulfate was initially added to 20% saturation, and the resulting precipitate was removed by centrifugation. The ammonium sulfate concentration in the supernatant was subsequently increased to 45% saturation, and the resulting immunoglobulin-containing precipitate was collected and dialyzed against PBS (pH 7.2). The resulting polyclonal antibody preparation was used as the primary antibody at a dilution of 1:20,000. HRP-conjugated goat anti-rabbit IgG (1:20,000; Sigma-Aldrich Chimie Sarl, Lyon, France) was used as the secondary antibody. Immunoreactive bands were detected using Clarity™ Max Western ECL substrate and visualized using a ChemiDoc MP Imaging System (Bio-Rad Laboratories, Hercules, CA, USA).

To assess N-glycosylation of rAppA-P, concentrated cell-free culture supernatants were treated with Endoglycosidase H (Endo H; New England Biolabs, Ipswich, MA, USA) according to the manufacturer’s instructions. Samples were incubated with Endo H at 37 °C for 16 h, with untreated samples processed in parallel as controls. Treated and untreated samples were subsequently analyzed using SDS-PAGE and Western blotting to assess changes in the apparent molecular weight of rAppA-P.

Protein molecular weights were estimated using Pierce™ Unstained Protein MW Marker (Thermo Scientific, Rockford, IL, USA; Cat. No. 26610) for SDS-PAGE and the Color Prestained Protein Standard (New England BioLabs, Ipswich, MA, USA; Cat. No. P7719S) for Western blotting.

### 2.7. Experimental Design and Shake-Flask Cultivation

Shake-flask cultivation was performed in 250 mL flat-bottom Erlenmeyer flasks containing 50 mL of YP medium (1% [*w*/*v*] yeast extract and 2% [*w*/*v*] peptone) supplemented with glucose at the concentrations specified below. The recombinant *K. phaffii* X-33 production strain described above was used throughout the experiments. Overnight cultures grown in YPD medium at 30 °C were used as inocula, and the experimental cultures were inoculated to an initial OD_600_ of 0.2.

The effects of cultivation temperature and initial glucose concentration on extracellular phytase production were evaluated using a 3 × 3 factorial design with three temperatures (24, 27, and 30 °C) and three initial glucose concentrations (1, 3, and 5% [*w*/*v*]), resulting in nine cultivation conditions. The temperature range was selected to compare the conventional cultivation temperature of 30 °C with moderately reduced temperatures. Each condition was evaluated using three independent biological replicates. The initial medium pH was adjusted to 7.0 and monitored during cultivation but was not controlled. All other cultivation parameters were maintained constant.

Cultures were incubated for 168 h on an orbital shaker at 200 rpm, and 3 mL samples were collected every 24 h to determine dry cell weight (DCW), residual glucose concentration, and extracellular phytase activity. Phytase activity was measured in cell-free culture supernatants obtained by centrifugation.

The cultivation condition yielding the highest extracellular phytase activity was selected for subsequent evaluation under controlled bioreactor conditions.

### 2.8. Phytase Activity Assay

Phytase activity of rAppA-E and rAppA-P was determined by measuring inorganic phosphate released from sodium phytate using a colourimetric assay [39]. The reaction mixture contained 0.1 mL of enzyme preparation and 0.9 mL of 2 mM sodium phytate in a 100 mM sodium acetate buffer (pH 4.5). After incubation at 65 °C for 10 min, the reaction was terminated by adding 0.75 mL of 5% (*w*/*v*) trichloroacetic acid (TCA). Released inorganic phosphate was quantified after adding 1.5 mL of the freshly prepared color reagent consisting of four volumes of 2.5% (*w*/*v*) (NH_4_)_2_MoO_4_ in 5.5% (*v*/*v*) H_2_SO_4_ and one volume of 2.5% (*w*/*v*) FeSO_4_. Absorbance was measured at 700 nm using a UV-1900i spectrophotometer (Shimadzu Corporation, Kyoto, Japan), and phosphate concentration was determined from a calibration curve prepared with KH_2_PO_4_. One unit of phytase activity (U) was defined as the amount of enzyme releasing 1 µmol of inorganic phosphate per minute under the assay conditions. The linearity of the assay with respect to reaction time and enzyme amount was experimentally verified under the standard assay conditions using rAppA-P preparations obtained from three independent cultivations (*n* = 3 biological replicates). Linearity with respect to reaction time was evaluated at 0, 2, 5, 7.5, and 10 min, including an experimentally measured zero-time control. Linear regression of the mean OD_700_ values yielded R^2^ = 0.992. Linearity with respect to enzyme amount was evaluated using 0.25-, 0.50-, 0.75-, and 1.00-fold amounts of the standard enzyme, and linear regression of the mean blank-corrected OD_700_ values yielded R^2^ = 0.996. The corresponding regression plots and individual replicate measurements are provided in Figure S1 and Table S2, respectively, in the Supplementary Materials.

Biomass-specific phytase activity was calculated as extracellular phytase activity (U/mL) divided by the corresponding dry cell weight concentration (g/L) and expressed as U/mg DCW.

### 2.9. Effect of pH on Phytase Activity

The effect of pH on rAppA-E and rAppA-P activity was evaluated over the 3.0–10.0 pH range at 0.5-unit intervals using the following 100 mM buffer systems: sodium acetate (pH 3.0–6.5), Tris-HCl (pH 7.0–8.5), and glycine–NaOH (pH 9.0–10.0). Sodium phytate (2 mM) was prepared in the corresponding buffer, and phytase activity was determined at 65 °C for 10 min as described in Section 2.8. Relative activity was expressed as a percentage of the maximum activity measured for each enzyme preparation.

### 2.10. Temperature–Activity Profile and Thermal Stability

The effect of temperature on rAppA-E and rAppA-P activity was evaluated by performing the phytase activity assay described in Section 2.7 at temperatures ranging from 30 to 90 °C at 5 °C intervals. Relative activity was expressed as a percentage of the maximum activity measured for each enzyme preparation.

Thermal stability was evaluated by preincubating the enzyme preparations without a substrate at 50, 55, 60, and 65 °C for rAppA-P and at 65, 70, 75, 80, and 85 °C for rAppA-E. Following preincubation, residual phytase activity was determined using the standard assay described in Section 2.8 and was expressed relative to the corresponding unheated control (100%). Short incubation intervals were included for rAppA-P due to its rapid thermal inactivation at elevated temperatures.

Thermal inactivation was analyzed using first-order regression of ln(A_t_/A_0_) against time, with the intercept fixed at zero, where A_t_ is the residual activity after preincubation for time t, and A_0_ is the activity of the corresponding unheated control. The apparent inactivation rate constant (k_d_) was calculated as the negative of the fitted slope, and the apparent half-life (t_1_/_2_) was calculated as ln(2)/k_d_. Zero-activity points were excluded from the regression because their logarithms are undefined. The goodness of fit was evaluated using the uncentered coefficient of determination (R^2^), which is appropriate for regression through the origin. The model was fitted separately to each of the three independent biological replicates. The resulting k_d_ and t_1/2_ estimates are reported as mean ± SD (*n* = 3).

### 2.11. Determination of Biomass

Dry cell weight (DCW) was determined gravimetrically. Culture samples were centrifuged at 5000× *g* for 20 min at 4 °C, and the cell pellets were washed once with distilled water. The washed pellets were transferred to pre-weighed aluminum dishes and dried at 80 °C to constant weight. DCW was calculated from the difference between the final and empty dish weights and expressed as g/L of culture.

### 2.12. Determination of Residual Glucose

Residual glucose concentration in cell-free culture supernatants was determined using the Vital Glucose enzymatic assay kit (Vital Development Corporation, St. Petersburg, Russia) according to the manufacturer’s instructions. Absorbance was measured using a UV-1900i spectrophotometer (Shimadzu, Japan), and glucose concentrations were expressed as g/L.

### 2.13. Bioreactor Cultivation

Batch cultivation was performed in a 2 L stirred-tank bioreactor (HABITAT, IKA-Werke GmbH & Co. KG, Staufen, Germany) under the cultivation conditions yielding the highest extracellular phytase activity in the shake-flask experiments (27 °C and 3% [*w*/*v*] initial glucose). Dissolved oxygen (DO) was continuously monitored using a DO probe and maintained at ≥30% of air saturation by automatic regulation of aeration. pH was not monitored or actively controlled during cultivation.

For inoculum preparation, the recombinant *K. phaffii* X-33 production strain was cultivated in 250 mL of YPD medium in flat-bottom flasks at 27 °C and 200 rpm. The inoculum volume was adjusted to obtain an initial bioreactor OD_600_ of 0.2. The production medium consisted of YP medium supplemented with 3% (*w*/*v*) glucose. The basal medium was sterilized by autoclaving at 121 °C for 20 min, whereas glucose was filter-sterilized through a 0.22 µm membrane and added aseptically.

Batch cultivation was conducted for 168 h, with 10 mL samples collected every 24 h to determine DCW, residual glucose concentration, and extracellular phytase activity as described above. Bioreactor cultivation comprised a single independent fermentation run, with each parameter measured in technical triplicate at each sampling time.

### 2.14. Statistical Analysis

Shake-flask data are presented as mean ± standard deviation (SD) from three independent biological replicates per cultivation condition. Dry cell weight and extracellular phytase activity were analyzed separately using linear mixed-effects models fitted by restricted maximum likelihood with the *nlme* package (version 3.1-169) in R (version 4.6.1; R Foundation for Statistical Computing, Vienna, Austria). Cultivation temperature, initial glucose concentration, and cultivation time were treated as categorical fixed effects, including all two- and three-way interactions. The cultivation flask was included as a random intercept. The activity model allowed residual variance to differ among temperature–glucose combinations, whereas the DCW model incorporated a second-order autoregressive residual correlation structure across sampling times within each flask. Covariance structures were selected based on residual diagnostics and Akaike’s information criterion. Fixed effects were evaluated using marginal F-tests with sum-to-zero contrasts, and estimated marginal means were obtained using the *emmeans* package (version 2.0.4). Statistical significance was defined as *p* < 0.05.

Residual glucose concentrations were summarized descriptively because the predominance of zero values was inconsistent with the assumptions of the Gaussian mixed-effects model. Biomass-specific phytase activity was calculated for each biological replicate and sampling time, as described in Section 2.8. Values were summarized as the mean ± SD of individual ratios. Bioreactor data represent a single independent cultivation run with three technical measurements per sampling time; therefore, the reported means and SDs reflect technical variation within this run rather than variation among independent fermentations. Comparisons between shake-flask and bioreactor cultivation were descriptive. 

## 3. Results

### 3.1. Expression of Recombinant AppA Phytases

**Figure 1:**
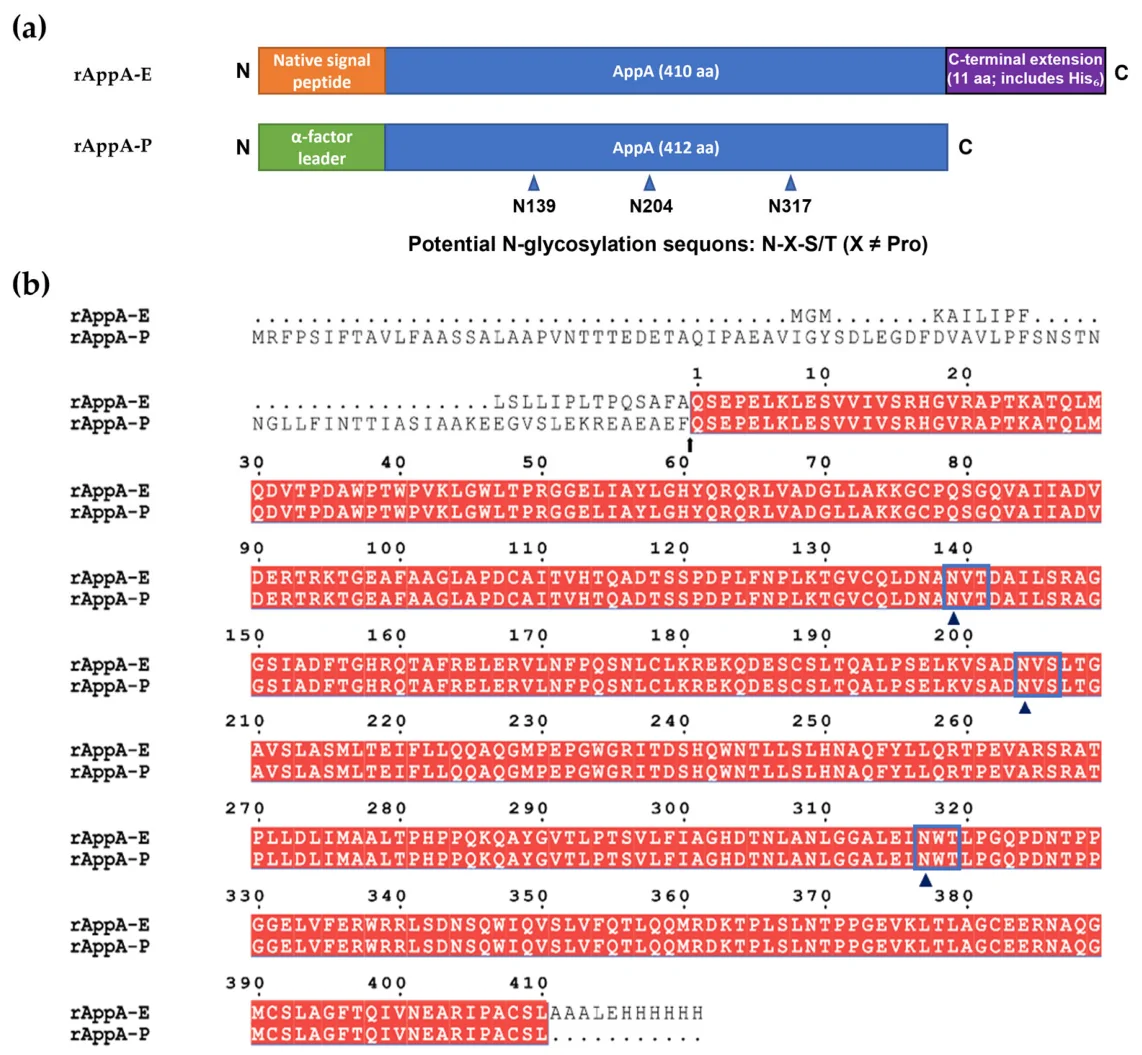
Schematic organization (**a**) and amino acid sequence alignment (**b**) of recombinant AppA variants produced in *E. coli* (rAppA-E) and *K. phaffii* (rAppA-P). Identical residues in the AppA region are highlighted in red; dots indicate alignment gaps. Blue triangles in (**a**) indicate the positions of the three predicted N-glycosylation sequons, while the corresponding sequons NVT (residues 139–141), NVS (204–206), and NWT (317–319) are highlighted by blue boxes in the sequence alignment in (**b**). Numbering starts at the N-terminal Gln of the 410-residue AppA region (Q1) and excludes the vector-derived terminal extensions. Panel (**a**) is not drawn to scale.

For comparative biochemical characterization, recombinant AppA phytases produced in *E. coli* and *K. phaffii* are referred to as rAppA-E and rAppA-P hereafter. The organization and amino acid sequence alignment of the recombinant variants are presented in Figure 1. Both variants share an identical 410-residue AppA region beginning with QSEPEL but differ in their N-terminal secretion sequences and C-terminal extensions. The bacterial construct encodes the native AppA signal peptide and a C-terminal extension, AAALEHHHHHH, containing the His_6_ tag. Following the removal of the N-terminal signal sequence, the predicted rAppA-E product comprises 421 residues, with a calculated molecular mass of approximately 45.9 kDa. The yeast construct encodes the *Saccharamyces cerevisiae* α-factor secretion signal followed by the vector-derived EF residues introduced at the *Eco*RI cloning junction. Following the expected Kex2/Ste13 processing of the α-factor leader, the predicted secreted rAppA-P retains the two N-terminal EF residues preceding the 410-residue AppA sequence. Thus, the predicted nonglycosylated rAppA-P comprises 412 residues and a calculated molecular mass of approximately 45.0 kDa.

Sequence analysis identified three potential N-glycosylation sequons in the shared AppA region: Asn139-Val140-Thr141 (NVT), Asn204-Val205-Ser206 (NVS), and Asn317-Trp318-Thr319 (NWT) (Figure 1). Residue numbering starts at the N-terminal Gln of the 410-residue AppA region and excludes the terminal extensions. These sequence motifs indicate potential glycosylation sites; individual occupancy was not determined experimentally.

**Figure 2:**
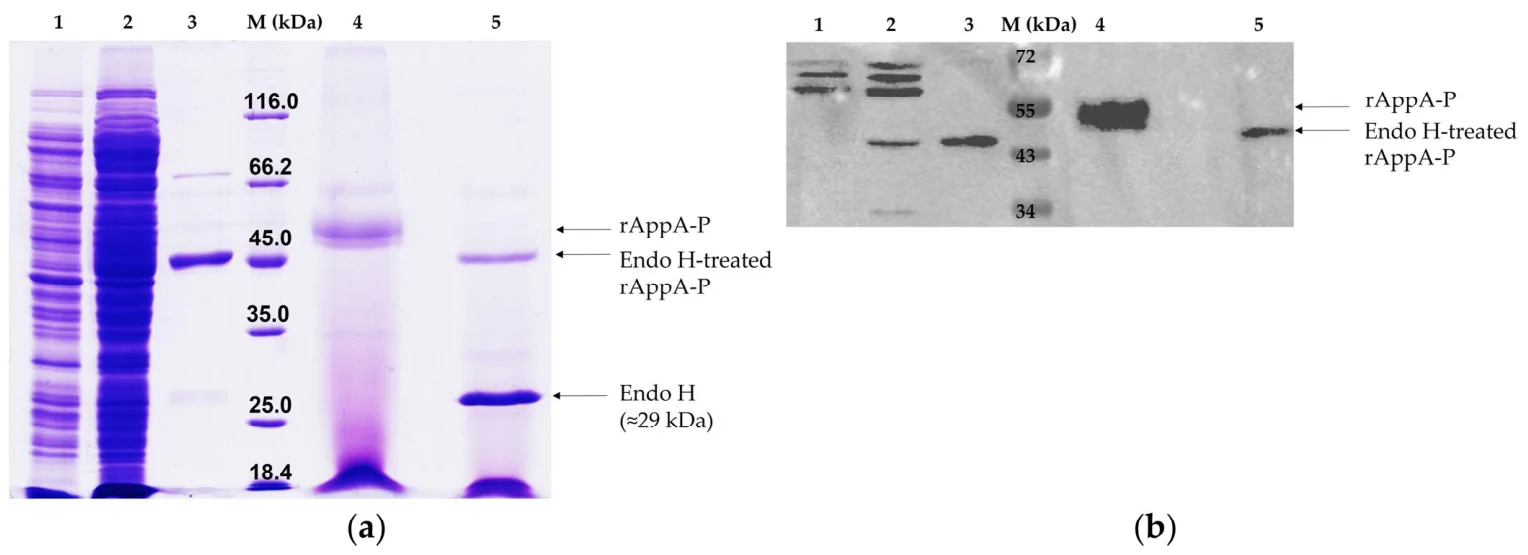
SDS-PAGE (**a**) and Western blot (**b**) analysis of recombinant AppA phytases produced in *E. coli* and *K. phaffii*. Lanes 1 and 2, *E. coli* cell lysates before and after IPTG induction, respectively; lane 3, purified rAppA-E; lane 4, 10-fold concentrated, 0.22 μm filtered cell-free *K. phaffii* culture supernatant containing rAppA-P; lane 5, the same preparation after Endo H treatment; M, molecular-weight marker (kDa). Different molecular-weight markers were used for SDS-PAGE (**a**) and Western blotting (**b**), as described in Section 2.6. The original images can be found in File S1.

The recombinant *K. phaffii* X-33 strain harbouring the integrated *appA* expression cassette derived from the pGAPZαA-*appA* vector secreted rAppA-P into the culture medium. SDS-PAGE analysis of the 10-fold concentrated, filtered cell-free culture supernatant revealed a predominant band with an apparent molecular mass of approximately 54 kDa (Figure 2a, lane 4), exceeding the calculated mass of the predicted nonglycosylated product. Western blot analysis using in-house rabbit polyclonal antibodies raised against recombinant AppA detected a corresponding immunoreactive band, supporting its identification as rAppA-P (Figure 2b, lane 4).

Treatment with Endo H shifted the apparent molecular mass of rAppA-P from approximately 54 to 45 kDa (Figure 2a,b, lane 5), close to the calculated value of 45 kDa for the predicted nonglycosylated form. This shift supports the presence of Endo H-sensitive N-linked glycans on rAppA-P; however, it does not establish which potential sites were modified or demonstrate complete removal of all carbohydrate residues. The treated protein also migrated near purified rAppA-E, which has a calculated molecular mass of approximately 45.9 kDa.

The preparations used for subsequent biochemical characterization differed: rAppA-E was affinity-purified, whereas rAppA-P was examined in an unconcentrated cell-free culture supernatant. The 10-fold concentrated and Endo H-treated rAppA-P samples were used only for protein analysis. Accordingly, biochemical comparisons concern these different preparations, and any observed differences cannot be attributed specifically to N-glycosylation.

### 3.2. Biochemical Characterization of Recombinant Phytase

**Figure 3:**
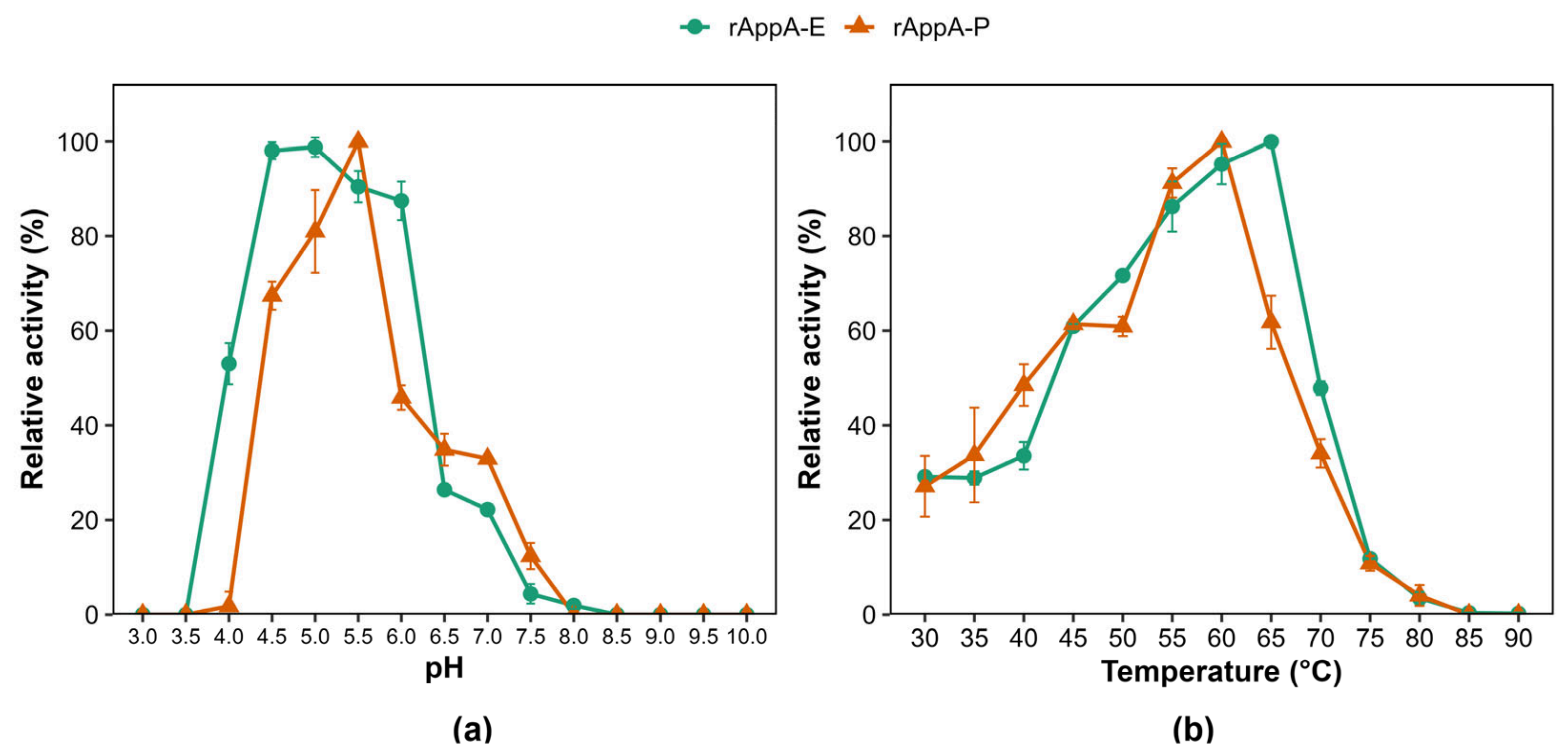
Effect of pH and temperature on the activity of recombinant AppA phytase preparations. (**a**) pH–activity profiles of rAppA-E and rAppA-P over the 3.0–10.0 pH range. (**b**) Temperature–activity profiles of rAppA-E and rAppA-P over the 30–90 °C temperature range. Relative activity was calculated separately for each enzyme preparation, with the maximum activity identified within each profile defined as 100%. Data are presented as mean ± SD from three independent biological replicates.

The recombinant phytases displayed distinct pH activity profiles (Figure 3a). rAppA-E maintained high relative activity over a broad pH range (4.5–6.0), whereas rAppA-P exhibited a narrower optimum with maximal activity at pH 5.5.

Temperature-dependent activity profiles also differed between the recombinant phytases (Figure 3b). The activity of rAppA-E increased gradually with temperature, reaching a maximum at 65 °C. In contrast, rAppA-P exhibited a sharper activity peak at 60 °C, followed by a pronounced decline at 65 °C. Despite these differences, both enzymes retained more than 60% of their maximal activity over the 45–65 °C range.

**Figure 4:**
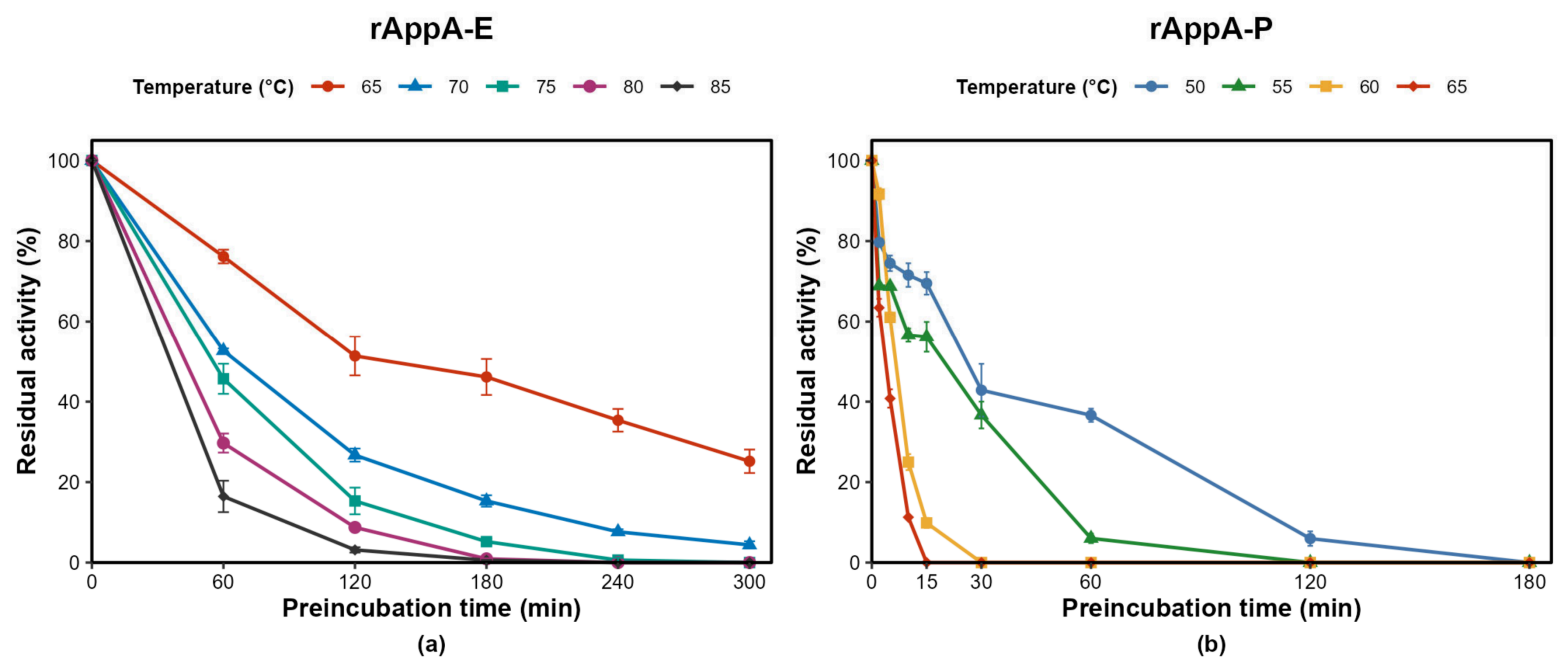
Thermal stability of recombinant AppA phytase preparations. (**a**) Residual activity of rAppA-E following preincubation at 65, 70, 75, 80, and 85 °C. (**b**) Residual activity of rAppA-P following preincubation at 50, 55, 60, and 65 °C. Enzyme preparations were preincubated without substrate for the indicated times, after which residual phytase activity was determined under the standard assay conditions. Residual activity was expressed relative to the corresponding unheated control (0 min), defined as 100%. Data are presented as mean ± SD from three independent biological replicates.

The thermostability profiles of rAppA-E and rAppA-P are shown in Figure 4a and b, respectively. Measurements at shorter preincubation intervals revealed the rapid loss of rAppA-P activity experienced at 65 °C: residual activity was approximately 63%, 41%, and 11% after 2, 5, and 10 min, respectively, and the measured activity was zero after 15 min (Figure 4b). For rAppA-E at 85 °C, the measured residual activity was zero after 240 and 300 min (Figure 4a).

Residual activity of rAppA-E declined progressively with increasing incubation time, with the inactivation rate strongly depending on temperature. The highest stability was observed at 65 °C, where approximately 25% of the initial activity remained after 300 min. At 70 °C, residual activity declined to below 5% over the same period. Further temperature increases markedly accelerated thermal inactivation, with almost complete loss of activity after 180–240 min at 75 and 80 °C.

The estimated apparent thermal inactivation rate constants (k_d_) and half-lives (t_1_/_2_) are summarized in Table 1. The apparent half-life of rAppA-P decreased from 30.51 ± 2.27 min at 50 °C to 3.28 ± 0.05 min at 65 °C, whereas that of rAppA-E decreased from 150.35 ± 5.08 min at 65 °C to 23.74 ± 0.96 min at 85 °C. At the common preincubation temperature of 65 °C, the apparent half-life was approximately 46-fold longer for rAppA-E than for rAppA-P (Table 1). Values are presented as mean ± SD from three independent biological replicates (*n* = 3).

### 3.3. Effects of Cultivation Temperature and Initial Glucose Concentration on Shake-Flask Cultivation

As the primary objective of this study was to optimize constitutive, methanol-free AppA production in *K. phaffii*, subsequent cultivation experiments focused on rAppA-P. rAppA-E was used as a bacterial reference for comparative biochemical characterization rather than the target of the production optimization study.

#### 3.3.1. Biomass Production and Glucose Consumption

**Figure 5:**
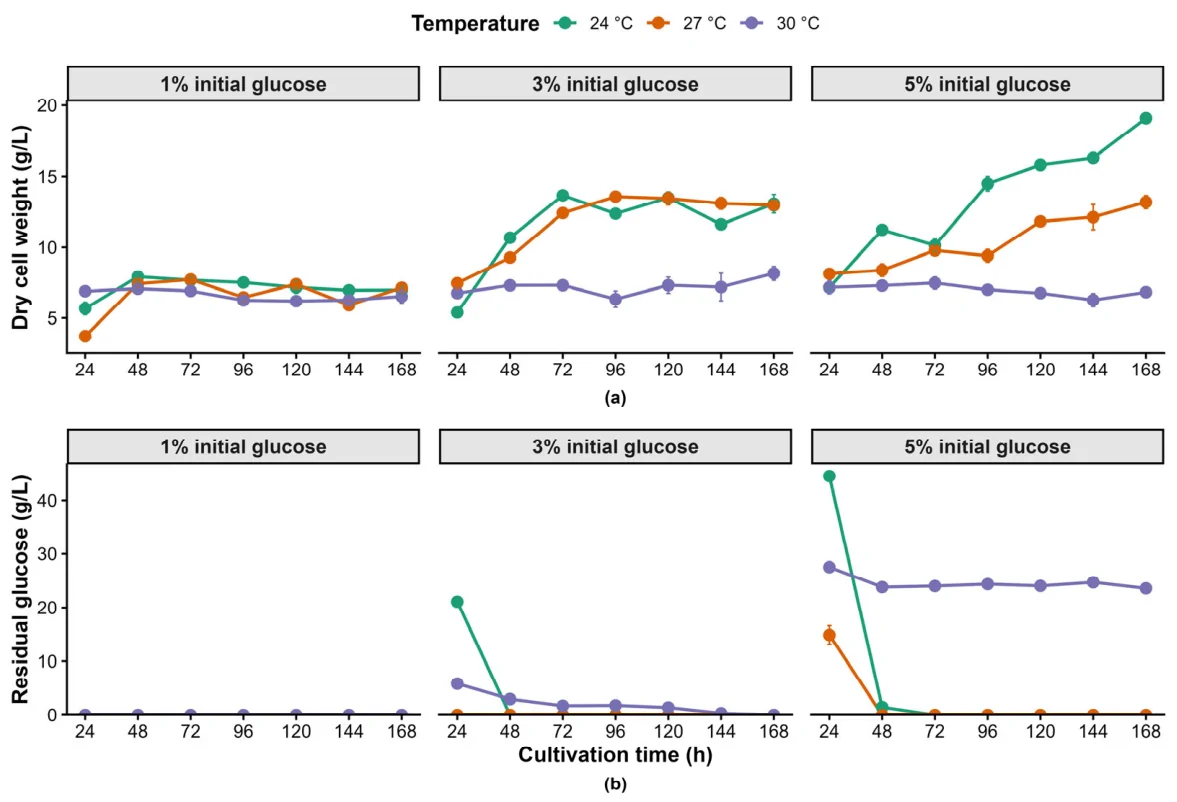
Biomass production and glucose consumption during shake-flask cultivation of recombinant *K. phaffii* X-33 expressing *appA* under different cultivation conditions. (**a**) Dry cell weight and (**b**) residual glucose concentration during cultivation at 24, 27, and 30 °C with initial glucose concentrations of 1%, 3%, and 5%. Data are presented as mean ± SD from three independent biological replicates.

Biomass accumulation varied substantially depending on cultivation temperature, initial glucose concentration, and cultivation time (Figure 5a). Increasing the initial glucose concentration promoted biomass accumulation at both 24 and 27 °C, whereas growth at 30 °C remained limited regardless of glucose availability. The highest dry cell weight (approximately 19 g/L) was achieved after 168 h of cultivation at 24 °C in medium containing 5% glucose. At 3% glucose, cultures grown at 24 and 27 °C reached comparable biomass concentrations of approximately 13–14 g/L, while cultivation with 1% glucose resulted in substantially lower biomass and showed little further increase after 48–72 h under all temperature conditions.

Glucose consumption showed a pronounced temperature- and concentration-dependent pattern (Figure 5b). Media containing 1% glucose were completely depleted by 24 h at all cultivation temperatures. At an initial glucose concentration of 3%, glucose was already depleted by 24 h at 27 °C, whereas at 24 °C, approximately 3 g/L remained at 48 h before declining to near-zero levels during subsequent cultivation. At 30 °C, glucose utilization was slower, with residual glucose decreasing gradually throughout cultivation. The most significant temperature-dependent differences were observed at an initial glucose concentration of 5%. At 24 and 27 °C, glucose was almost completely consumed by 48 h, whereas at 30 °C, approximately 24–25 g/L residual glucose was maintained throughout most of the cultivation period. Thus, limited biomass accumulation occurred at 30 °C despite continued glucose availability. The culture pH decreased from the initial value of 7.0 to 4.67 ± 0.05 during the first 24 h and subsequently remained relatively stable throughout cultivation.

#### 3.3.2. Extracellular Phytase Production

**Figure 6:**
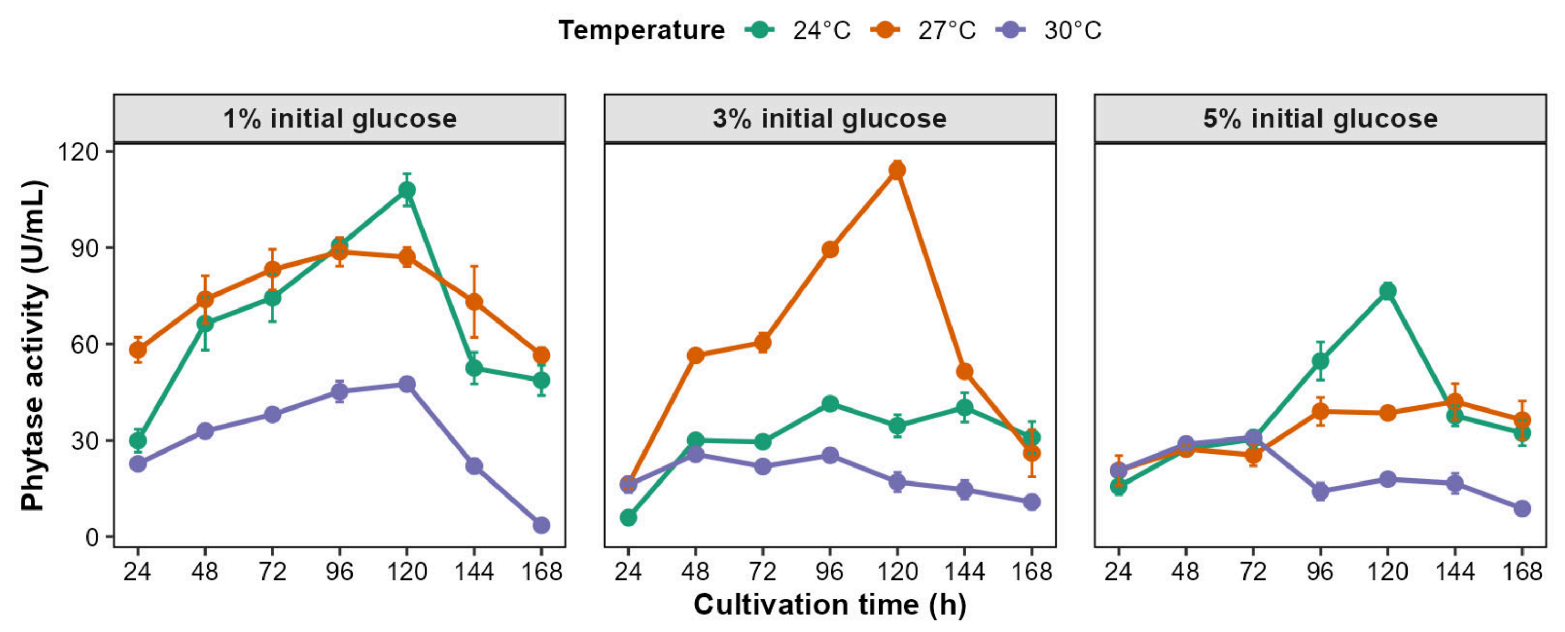
Extracellular rAppA-P phytase activity during shake-flask cultivation.

Extracellular phytase production varied significantly based on cultivation temperature, initial glucose concentration, and cultivation time (Figure 6). In contrast to biomass accumulation, maximal phytase production was not achieved under the conditions of highest cell growth. The highest extracellular phytase activity (114.17 ± 2.71 U/mL) was obtained after 120 h of cultivation at 27 °C in medium containing 3% glucose.

At 1% glucose, phytase production remained relatively high over a broad cultivation period, although the temporal production profiles differed across temperatures. At 24 °C, enzyme activity increased steadily and reached a maximum after 120 h regardless of the initial glucose concentration. In contrast, cultures grown at 27 °C exhibited earlier phytase production at 1% glucose, with peak activity reached after 96 h; by contrast, maximal activity at 3% and 5% glucose was observed after 120 h. Cultivation at 30 °C consistently resulted in lower phytase activities, followed by a rapid decline after the activity peak was reached.

Increasing the initial glucose concentration from 3% to 5% did not improve extracellular phytase production despite promoting biomass accumulation. At 5% glucose, phytase activity remained comparatively low under all temperature conditions except at 24 °C, where enzyme production increased markedly after 72 h and reached its maximum at 120 h.

#### 3.3.3. Biomass-Specific Phytase Activity

**Figure 7:**
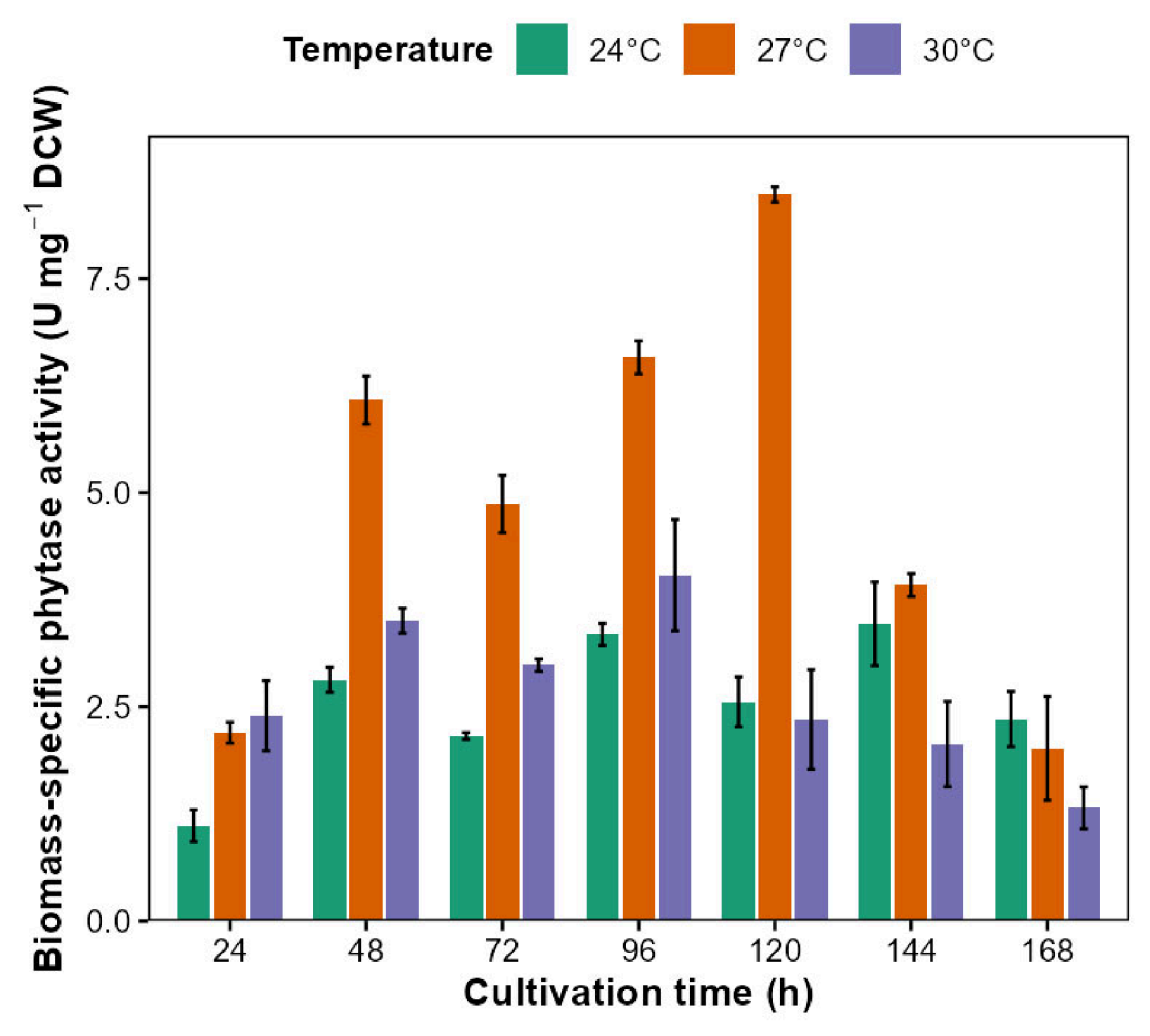
Biomass-specific rAppA-P phytase activity during shake-flask cultivation at different temperatures with an initial glucose concentration of 3% (*w*/*v*). Data are presented as mean ± SD.

To distinguish between biomass accumulation and enzyme production, extracellular phytase activity was normalized to dry cell weight. Because cultivation with 3% initial glucose yielded the highest volumetric phytase activity and was subsequently selected for bioreactor cultivation, biomass-specific phytase activity under this condition was examined in greater detail (Figure 7). The temporal profiles differed considerably among cultivation temperatures, indicating that enzyme production per unit biomass changed over time.

At 27 °C, biomass-specific phytase activity was generally higher than at 24 and 30 °C. It increased from approximately 2.2 U/mg DCW at 24 h to 6.1 U/mg DCW at 48 h, decreased temporarily at 72 h, and subsequently increased to approximately 6.6 U/mg DCW at 96 h. The highest value, approximately 8.5 U/mg DCW, was reached after 120 h, followed by a pronounced decline during prolonged cultivation.

At 24 °C, biomass-specific phytase activity increased more gradually and reached a maximum of approximately 3.5 U/mg DCW at 144 h. At 30 °C, the specific activity increased to approximately 4.0 U/mg DCW at 96 h and subsequently declined. Thus, among the three temperatures examined at 3% initial glucose, cultivation at 27 °C yielded the highest biomass-specific phytase activity, with the maximum observed after 120 h.

Linear mixed-effects analysis revealed significant effects of cultivation temperature, initial glucose concentration, and cultivation time on dry cell weight and extracellular phytase activity. All two-way interactions and the three-way interaction were significant for both response variables (all *p* < 0.001), indicating that the effects of cultivation conditions varied over time and depended on the combination of temperature and initial glucose concentration.

### 3.4. Scale-Up of Recombinant Phytase Production in a 2 L Bioreactor

**Figure 8:**
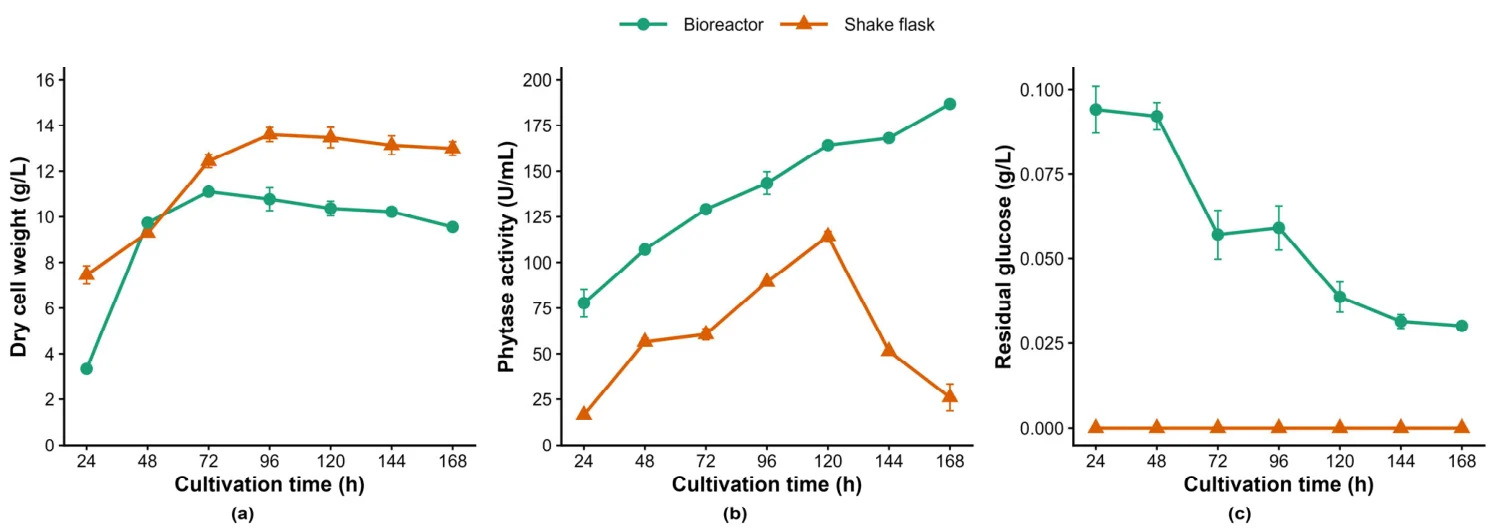
Comparison of shake-flask and 2 L stirred-tank bioreactor cultivation at 27 °C with an initial glucose concentration of 3% (*w*/*v*). (**a**) Dry cell weight; (**b**) extracellular rAppA-P phytase activity; (**c**) residual glucose concentration. Shake-flask values are the mean ± SD from three independent biological replicates. Bioreactor values are the mean ± SD from three technical measurements per sampling time in one independent cultivation run. The comparison is descriptive.

Batch fermentation was performed in a 2 L stirred-tank bioreactor under the cultivation conditions selected during shake-flask optimization (27 °C, 3% initial glucose). Cell growth, residual glucose concentration, and extracellular phytase production were monitored throughout the 168 h cultivation period. Biomass accumulation, glucose utilization, and phytase production in the bioreactor were compared with those obtained during shake-flask cultivation under the same temperature and initial glucose concentration (Figure 8).

Biomass increased rapidly during the first 72 h of bioreactor cultivation, reaching a maximum dry cell weight of 11.11 ± 0.14 g/L at 72 h (Figure 8a). Thereafter, biomass gradually decreased and reached 9.55 ± 0.07 g/L at 168 h. In shake-flask cultures, biomass accumulation followed a different pattern, reaching higher values during the later stages of cultivation and remaining at approximately 13 g/L from 96 to 168 h (Figure 8a). Glucose utilization also differed between the two cultivation systems (Figure 8c). In shake-flask cultures, no residual glucose was detected from 24 h onward, indicating that available glucose was already depleted at the first sampling point. In contrast, a low but measurable residual glucose concentration remained throughout bioreactor cultivation, decreasing from 0.094 ± 0.007 g/L at 24 h to 0.030 ± 0.001 g/L at 168 h. Thus, although most of the glucose was consumed during the first 24 h in the bioreactor, complete depletion was not observed during the subsequent cultivation period.

In contrast to biomass accumulation, the bioreactor’s extracellular phytase activity increased continuously throughout the cultivation period (Figure 8b). Activity increased from 77.68 ± 7.58 U/mL at 24 h to 129.13 ± 2.20 U/mL at 72 h and reached a maximum of 186.81 ± 0.25 U/mL at 168 h. A markedly different production profile was observed in shake-flask cultures: phytase activity increased to a maximum of 114.17 ± 2.71 U/mL at 120 h and subsequently declined (Figure 8b). Thus, while shake-flask cultivation resulted in greater biomass accumulation during the later stages of cultivation, controlled bioreactor cultivation supported continued accumulation of extracellular phytase despite the decline in biomass and very low residual glucose concentration.

In this single bioreactor run, the maximum extracellular phytase activity (186.81 ± 0.25 U/mL) was approximately 1.6-fold higher than the maximum mean value obtained in shake-flask cultures (114.17 ± 2.71 U/mL). Additional independent bioreactor runs are required to establish the reproducibility of this difference.

## 4. Discussion

Constitutive expression under the GAP promoter enabled methanol-free production of recombinant AppA phytase in *K. phaffii*. As previously shown, GAP-driven expression can constitutively produce other secreted recombinant enzymes in this host, including camel prochymosin B production [34]. Alternative methanol-free expression strategies have also been developed in this host, including alkaline-pH-regulatable promoter systems to produce industrial enzymes [40]. Among the relatively few studies describing constitutive AppA expression in this host, Tai et al. [31] reported an extracellular phytase activity of 112.5 U/mL in shake-flask cultivation, comparable to the maximum activity obtained in the present study. Higher phytase titres have been reported under AOX1 control, particularly during high-cell-density fed-batch cultivation with methanol induction [41]. More recently, Luna-Flores et al. [32] demonstrated the importance of bioprocess optimization for phytase production in *K. phaffii* using a strategy combining de-repression with subsequent methanol induction. These studies provide context for further development of constitutive AppA production through cultivation optimization. In the present study, transfer to bioreactor cultivation was associated with higher extracellular activity in a single run; independent cultivations are required to assess the reproducibility of this increase. The relevance of rAppA-P therefore lies in its potential for constitutive, methanol-free extracellular production rather than in superior biochemical performance relative to rAppA-E.

The *S. cerevisiae* α-mating factor directed the secretion of rAppA-P into the culture medium, where it became the predominant extracellular protein. Extracellular production is a practical advantage of *K. phaffii* because it avoids cell disruption and can simplify subsequent downstream processing [24,42,43]. Gratzer et al. illustrated the importance of secretion efficiency for recombinant phytase production [44] by investigating thermostable phytase secretion in *K. phaffii*. In the present study, however, rAppA-P was characterized directly in the cell-free culture supernatant, whereas rAppA-E was analyzed after affinity purification. This difference in preparation should be considered when interpreting the biochemical comparison, because culture-medium components and other extracellular proteins may influence apparent enzyme activity and stability.

Endo H treatment reduced the apparent molecular weight of rAppA-P from approximately 54 to 45 kDa, supporting N-glycosylation of the yeast-derived enzyme. Glycosylation of recombinant proteins produced in *K. phaffii* can affect secretion, molecular properties, and biochemical behaviour; however, the magnitude and direction of these effects are strongly dependent on the target protein, the glycosylation site, and the expression context [45,46,47,48,49,50]. Consistent with this complexity, rAppA-E and rAppA-P exhibited different pH– and temperature–activity profiles despite being derived from the same *appA* coding sequence. However, because purified glycosylated and deglycosylated rAppA-P were not compared, alongside identically purified preparations from both hosts, the observed biochemical differences cannot be attributed specifically to N-glycosylation.

The most significant difference between the two preparations concerned thermal behaviour. rAppA-P showed substantially lower apparent thermostability than purified rAppA-E. Although glycosylation is often associated with increased protein stability, its effects are not universally stabilizing and depend on the local structural context [46,50]. Several mechanisms could contribute to the behaviour observed for rAppA-P. Heterogeneity in glycan structure or site occupancy may contribute to differences in protein conformation and stability, while the steric effects of carbohydrate chains may further influence protein behaviour [51,52]. In addition, host-dependent differences in protein folding, disulfide-bond formation, or proteolytic processing during recombinant protein production in *K. phaffii* may also affect the properties of the resulting protein [53]. These possibilities were not directly examined and therefore remain hypotheses rather than demonstrated causes. Moreover, because rAppA-E and rAppA-P differed in purification state, their thermal-stability profiles should be interpreted as properties of the respective experimental preparations rather than a direct measure of the effect of glycosylation or expression host alone. From an application perspective, the lower apparent thermostability observed for rAppA-P preparation indicates that thermal robustness warrants further investigation and development.

Cultivation conditions affected recombinant phytase production differently from biomass formation. Increasing the initial glucose concentration generally promoted biomass accumulation, but this increase did not translate proportionally into extracellular phytase production. At 24 and 27 °C, glucose was largely depleted early in cultivation while biomass continued to increase, whereas at 30 °C a considerable amount of residual glucose remained present despite comparatively limited biomass formation. Thus, the reduced growth observed at 30 °C cannot be explained by carbon depletion alone. More generally, the divergence between biomass accumulation and extracellular enzyme activity indicates that conditions favouring rapid cell growth do not necessarily favour recombinant protein production. Similar uncoupling between biomass formation and heterologous protein production has been reported for *K. phaffii*, particularly when carbon availability and cellular metabolic state affect secretory capacity [50,51].

Cultivation temperature showed a comparable trade-off. Although approximately 30 °C is commonly used for *K. phaffii* growth, the moderately reduced temperatures included in the present experimental design were intended to evaluate recombinant phytase production rather than to identify the optimum temperature for yeast biomass formation. Extracellular phytase production was more favourable at 24 and 27 °C than at 30 °C under the conditions tested. Reduced cultivation temperatures have also been reported to improve production of other recombinant proteins in *K. phaffii* [54,55,56], potentially by reducing cellular stress and the burden on the secretory pathway and allowing more efficient folding and maturation of heterologous proteins. Although not directly investigated here, these mechanisms provide a plausible physiological basis for the divergence between conditions favouring biomass formation and those favouring extracellular phytase accumulation.

This distinction is also important when defining the most favourable cultivation condition. The 27 °C/3% glucose condition was selected for subsequent bioreactor cultivation because it produced the highest volumetric extracellular phytase titre among the shake-flask conditions tested. It should therefore not be interpreted as representing a universal process optimum. The present study examined cultivation temperature and initial glucose concentration while keeping the other cultivation conditions constant. Further systematic evaluation of medium composition, including nitrogen source and carbon-to-nitrogen ratio, together with cultivation pH and oxygen supply, is therefore required for more comprehensive process optimization. Lower glucose availability produced substantially higher biomass-specific activity under some conditions, including approximately 15.9 U/mg DCW at 27 °C with 1% glucose after 24 h of cultivation and approximately 15.1 U/mg DCW at 24 °C with 1% glucose after 120 h of cultivation. Such conditions may be attractive when substrate utilization or biomass-specific activity is prioritized, while the high biomass-specific activity observed at 27 °C with 1% glucose after only 24 h also indicates potential relevance when a shorter cultivation time is considered. Conversely, 27 °C with 3% glucose is more appropriate when the primary objective is to reach maximum extracellular enzyme titre. This distinction emphasizes that process optimization depends on the performance criterion selected rather than on a single biological response.

Transfer of the 27 °C/3% glucose condition to a 2 L stirred-tank bioreactor demonstrated the feasibility of controlled cultivation of the constitutive rAppA-P-producing strain. Although no residual glucose was detected in the shake-flask cultures at 24 h, only 0.094 ± 0.007 g/L remained in the bioreactor at the same sampling time, indicating that the initial glucose was almost completely consumed in both cultivation systems within the first 24 h. Because 24 h was the first post-inoculation sampling point, the actual glucose-consumption kinetics during the initial 24 h cannot be resolved from the present data, and therefore the rates of glucose utilization in shake-flask and bioreactor cultures cannot be directly compared. Differences in the resulting biomass profiles should consequently not be interpreted solely on the basis of the residual glucose concentrations measured at 24 h. In this single bioreactor run, the maximum extracellular phytase activity was approximately 1.6-fold higher than the maximum mean value obtained in shake flasks. Controlled oxygen transfer, more homogeneous mixing, and improved mass transfer plausibly contribute to this difference, as oxygen availability influences cellular stress, respiratory metabolism, and recombinant protein secretion in *K. phaffii* [48,54,57,58]. However, the extent to which each factor contributes cannot be isolated, and the absence of independent replicate fermentations prevents conclusions about the reproducibility of the observed increase.

The late-stage bioreactor behaviour also requires cautious interpretation. Biomass declined after its maximum; by contrast, extracellular phytase activity continued to increase. Because pH was not actively controlled and cell viability and microscopic integrity were not evaluated, this pattern cannot be attributed unequivocally to continued secretion by viable cells. Continued extracellular production may have contributed; however, cell damage or lysis and release of intracellular or cell-associated enzymes cannot be excluded. Similarly, the decline in extracellular phytase activity during late shake-flask cultivation may reflect reduced enzyme stability or proteolytic degradation. Increased extracellular protease activity and release of intracellular or vacuolar proteases have been described during prolonged *K. phaffii* cultivation [58,59]; however, neither protease activity nor AppA degradation was directly measured in the present study. Future controlled fermentations should therefore include pH regulation and monitoring of viability and culture integrity to clearly distinguish production, release, and degradation processes.

The continued increase in extracellular phytase activity up to the final bioreactor sampling point suggests that the maximum titre may not have been reached within the 168 h cultivation period investigated. Therefore, cultivation beyond 168 h should be evaluated to determine whether extracellular phytase activity increases further or reaches a plateau. Such experiments should monitor substrate availability, pH, cell viability, and culture integrity to better distinguish continued enzyme production from possible effects associated with the late cultivation phase. At the same time, further process development should explore strategies aiming to achieve comparable or higher titres within a shorter cultivation period, including fed-batch carbon supply, pH regulation, and optimization of oxygen transfer [48,49]. Thus, both the potential benefit of extending cultivation and opportunities for process intensification warrant further investigation.

Thermal stability represents a second major target for further development. The present thermostability experiments were designed to characterize enzyme inactivation during prolonged exposure and do not reproduce the short-term, high-temperature stresses encountered during feed manufacture. Consequently, the observed loss of activity during prolonged heating should not be interpreted directly as resistance to pelleting or other short-term feed-processing conditions. Evaluating residual activity after brief high-temperature exposure, together with stabilization through protein engineering, glycoengineering, or formulation where required, would provide a more application-relevant assessment of rAppA-P.

Overall, the results demonstrate that constitutive GAP-driven expression can support substantial extracellular production of AppA in *K. phaffii* without methanol induction, while also revealing important trade-offs between biomass formation, volumetric enzyme production, and biomass-specific activity. The differences observed between rAppA-P and rAppA-E highlight the influence of expression host and experimental preparation on the apparent biochemical phenotype, but do not permit the effects of glycosylation to be isolated from other host- and preparation-dependent factors. Among the conditions tested, 27 °C with 3% glucose was most suitable when maximum extracellular phytase titre was the primary process objective, whereas lower glucose concentrations were advantageous in biomass-specific production. Together, these findings identify both controlled bioprocess development and improved thermal robustness as the principal directions for further optimization of constitutive rAppA-P production.

## 5. Conclusions

This study demonstrates the feasibility of constitutive, methanol-free production of *E. coli* AppA phytase in *K. phaffii* under GAP promoter control, showing that extracellular enzyme production varies with cultivation strategy. Importantly, the conditions favouring biomass accumulation, biomass-specific phytase activity, and maximum volumetric phytase production were not identical, highlighting the need to define the primary process objective when developing recombinant phytase production.

The combination of constitutive expression, extracellular secretion, and compatibility with controlled stirred-tank cultivation provides a basis for further development of rAppA-P production in *K. phaffii*. Under the conditions examined, the rAppA-P preparation showed lower thermal stability than purified rAppA-E. However, because rAppA-P was characterized in a cell-free culture supernatant rather than a purified enzyme, this difference cannot be attributed specifically to intrinsic enzyme properties or host-dependent glycosylation. Moreover, the thermal stability experiments performed in this study did not directly reproduce the short-term high-temperature exposure relevant to feed processing. Future studies should therefore characterize purified rAppA-P, including direct comparison of its glycosylated and deglycosylated forms, and evaluate short-term thermal resistance under feed-processing-relevant conditions. Further process development should include fed-batch cultivation, pH control, optimization of oxygen and carbon supply, and monitoring of cell viability and integrity during prolonged cultivation. Together, these studies can establish the intrinsic biochemical properties of rAppA-P and determine the potential of the constitutive *K. phaffii* system for efficient AppA production and future feed applications.

## Figures and Tables

**Table 1:** Apparent first-order thermal inactivation parameters of rAppA-E and rAppA-P.

Enzyme	Temperature (°C)	k_d_, Min^−1^, Mean ± SD	t_1/2_, Min, Mean ± SD
rAppA-P	50	0.02281 ± 0.00172	30.51 ± 2.27
rAppA-P	55	0.04463 ± 0.00237	15.56 ± 0.80
rAppA-P	60	0.14511 ± 0.00606	4.78 ± 0.20
rAppA-P	65	0.21111 ± 0.00314	3.28 ± 0.05
rAppA-E	65	0.00461 ± 0.00016	150.35 ± 5.08
rAppA-E	70	0.01058 ± 0.00029	65.53 ± 1.81
rAppA-E	75	0.01791 ± 0.00246	39.24 ± 5.82
rAppA-E	80	0.02536 ± 0.00217	27.47 ± 2.46
rAppA-E	85	0.02923 ± 0.00117	23.74 ± 0.96

## Data Availability

The data supporting the findings of this study are available from the corresponding author upon reasonable request.

## Supplementary Materials

The following supporting information can be downloaded at: https://www.mdpi.com/article/10.3390/biology15191758/s1, Figure S1: Linearity of the rAppA-P phytase assay with respect to reaction time and relative enzyme amount; Table S1: Oligonucleotide primers used in this study; Table S2: Individual OD_700_ readings used to evaluate assay linearity for rAppA-P at 65 °C. File S1.The original images of the western blots and SDS-PAGE.

## Abbreviations

The following abbreviations are used in this manuscript:

AOX1	Alcohol oxidase 1 promoter
AppA	*Escherichia coli* AppA phytase
DCW	Dry cell weight
DO	Dissolved oxygen
Endo H	Endoglycosidase H
GAP	Glyceraldehyde-3-phosphate dehydrogenase promoter
IPTG	Isopropyl β-D-1-thiogalactopyranoside
LB	Luria–Bertani medium
PVDF	Polyvinylidene difluoride
rAppA-E	Recombinant AppA phytase produced in *Escherichia coli*
rAppA-P	Recombinant AppA phytase produced in *Komagataella phaffii*
SDS-PAGE	Sodium dodecyl sulfate–polyacrylamide gel electrophoresis
YPD	Yeast extract–peptone–dextrose medium
