# Supplementary material for: Methanol-Free Production of Recombinant AppA Phytase in Komagataella phaffii: Evaluation of Cultivation Conditions, Bioreactor Cultivation, and Biochemical Characterization
Source: Biology (Basel). 2026 Oct 2;15(19):1758. doi: 10.3390/biology15191758 (PMC13664581; doi:10.3390/biology15191758)
Supplement: Supplementary file 1 [file biology-15-01758-s001.zip › biology-4526115-supplementary.pdf]

SUPPLEMENTARY MATERIALS

Methanol-Free Production of Recombinant AppA Phytase in *Komagataella phaffii*: Evaluation of Cultivation Conditions, Bioreactor Cultivation, and Biochemical Characterization

Table S1. Oligonucleotide primers used in this study.

Primer	Sequence (5′–3′)	Restriction site	Application
AppA-Efw	CATGCCATGGGGATGAAAGCGATCTTAATCCC	*Nco*I	Amplification of *appA* for bacterial expression
AppA-Erv	TTTTCCTTTTGCGGCCGCCAAACTGCACGCCGG	*Not*I	Amplification of *appA* for bacterial expression
T7fw	TAATACGACTCACTATAGGG	—	Screening of pET-28/*appA* constructs
T7rv	GCTAGTTATTGCTCAGCGG	—	Screening of pET-28/*appA* constructs
AppA-EcoRI	CCGGAATTCCAGAGTGAGCCTGAG	*Eco*RI	Amplification of codon-optimized *appA* for yeast expression
AppA-NotI	TTTTCCTTTTGCGGCCGCCTACAAGGAACAAGCTGGGA	*Not*I	Amplification of codon-optimized *appA* for yeast expression
GAPfw	GTCCCTATTTCAATCAATTGAA	—	Screening of pGAPZαA-*appA* and verification of genomic integration
AOX1rv	GCAAATGGCATTCTGACATCC	—	Screening of pGAPZαA-*appA* and verification of genomic integration

Verification of phytase assay linearity

Linearity was evaluated using rAppA-P preparations obtained from three independent cultivations (n = 3 biological replicates). Both experiments were conducted under the standard assay conditions at 65 °C. Figure S1 presents the measurements and regressions; Table S2 provides the individual OD_{700} readings.

Linearity with respect to reaction time was assessed over 0–10 min. The zero-time control was measured experimentally. The regression was fitted to the mean values at 0, 2, 5, 7.5, and 10 min. The experimentally measured OD_{700} values, including the zero-time control, were used directly for regression analysis without subtraction of the zero-time value.

For the enzyme-amount experiment, 25, 50, 75, and 100 μL of each 1:100-diluted preparation were used, corresponding to 0.25×, 0.50×, 0.75×, and 1.00× the standard enzyme amount. Buffer maintained the enzyme-plus-buffer volume at 100 μL, and the reaction time was 10 min. Regressions were fitted by ordinary least squares to the means, with freely estimated intercepts.

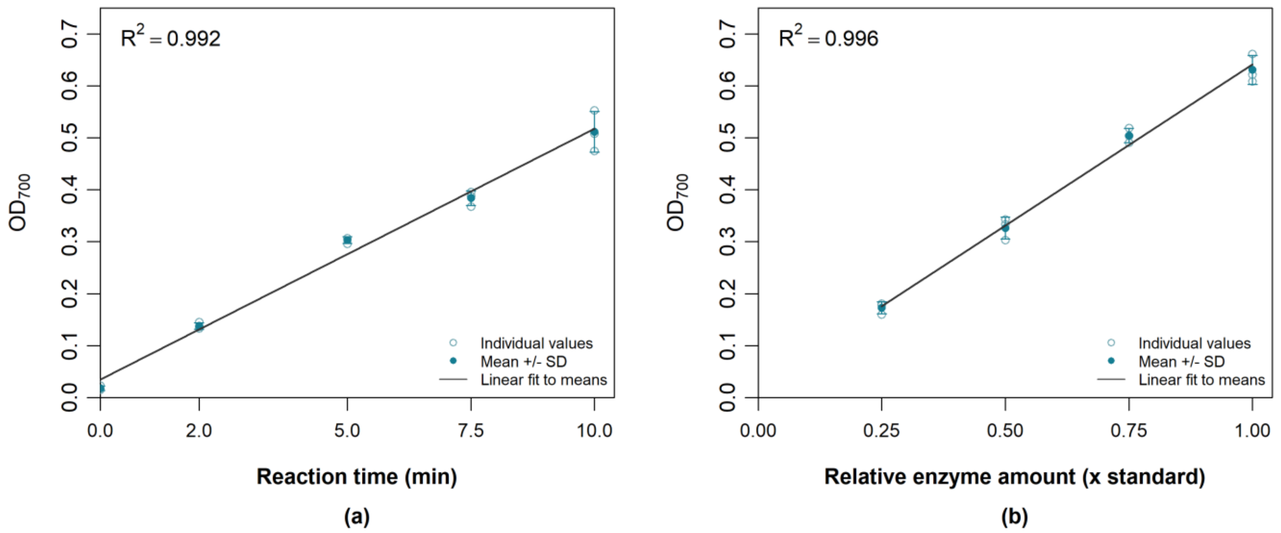

Figure S1. Linearity of the rAppA-P phytase assay with respect to (a) reaction time and (b) relative enzyme amount at 65 °C. Open circles show individual biological-replicate values; filled circles and error bars show mean ± SD (n = 3). Solid lines represent linear regressions of the means, fitted over 0–10 min in (a) and 0.25×–1.00× in (b). R^2 values are centered coefficients of determination from regressions with freely estimated intercepts.

The fitted equations were $OD_{700} = 0.048336t + 0.034218$ (t in min; $R^2 = 0.991799$) and $OD_{700} = 0.620800x + 0.020833$ (x is relative enzyme amount; $R^2 = 0.996067$).

Underlying measurements

Table S2. Individual OD_{700} readings used to evaluate assay linearity for rAppA-P at 65 °C.

(a) Reaction-time series

Time (min)	Replicate 1	Replicate 2	Replicate 3	Mean	SD
0	0.015	0.016	0.023	0.0180	0.0044
2	0.133	0.136	0.145	0.1380	0.0062
5	0.307	0.296	0.307	0.3033	0.0064
7.5	0.396	0.388	0.368	0.3840	0.0144
10	0.553	0.508	0.475	0.5120	0.0392

All five time points in the 0–10 min validation interval were included in the regression.

(b) Enzyme-amount series

Relative amount	Replicate 1	Replicate 2	Replicate 3	Mean	SD
0.25	0.181	0.160	0.178	0.1730	0.0114
0.5	0.303	0.334	0.343	0.3267	0.0210
0.75	0.504	0.491	0.519	0.5047	0.0140
1	0.622	0.662	0.609	0.6310	0.0276

Each condition has three biological-replicate readings from independent cultivations. Replicate columns retain the order of values in the source tables. Means and sample SDs were calculated from the three readings. Enzyme-amount readings were blank-corrected prior to regression analysis. The displayed R^2 values were calculated from regressions fitted to the mean values across the three biological replicates.
