# Supplementary material for: Methanol-Free Production of Recombinant AppA Phytase in Komagataella phaffii: Evaluation of Cultivation Conditions, Bioreactor Cultivation, and Biochemical Characterization
Source: Biology (Basel). 2026 Oct 2;15(19):1758. doi: 10.3390/biology15191758 (PMC13664581; doi:10.3390/biology15191758)

Figure S1 – Figure 1A

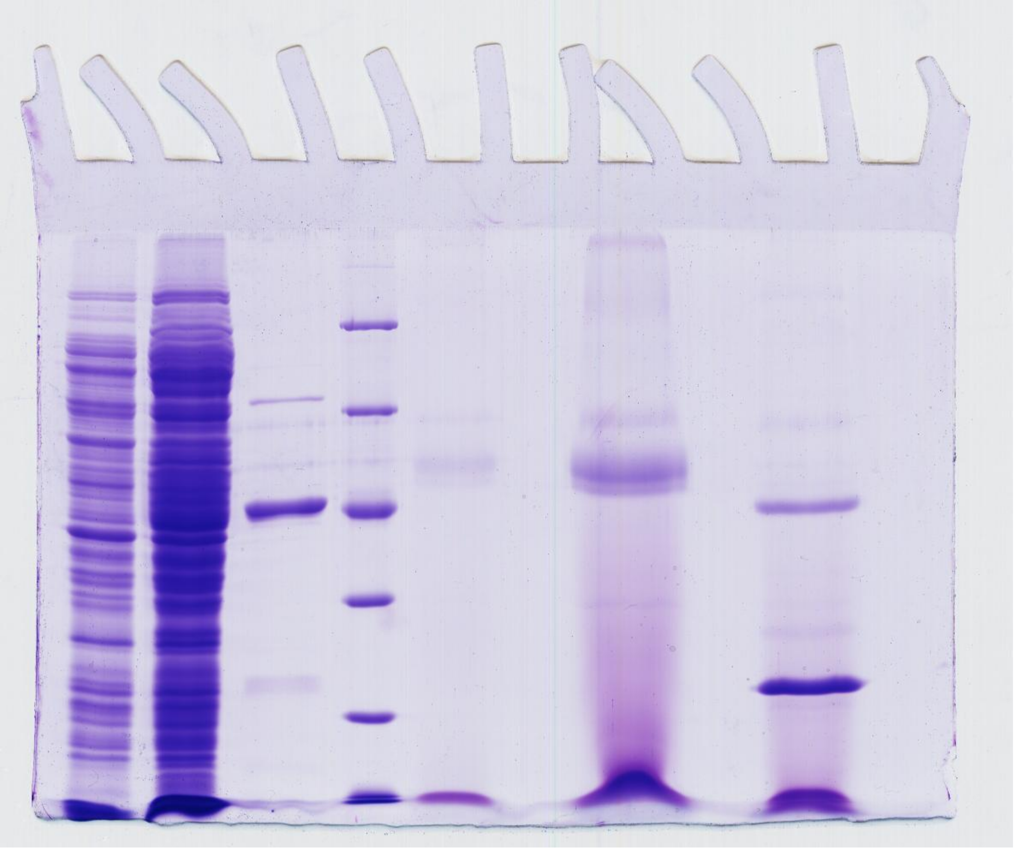

Figure S2 – Figure 1B

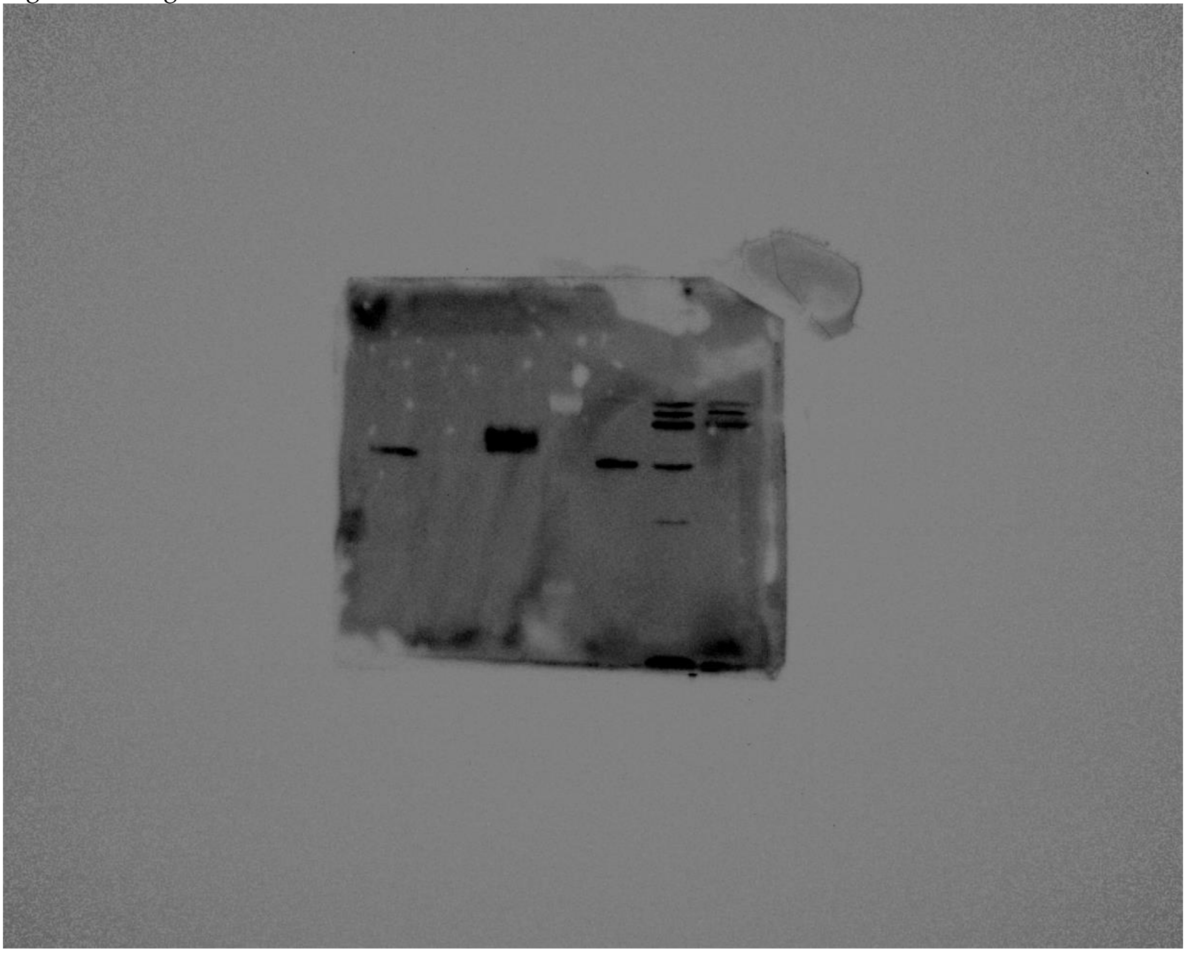

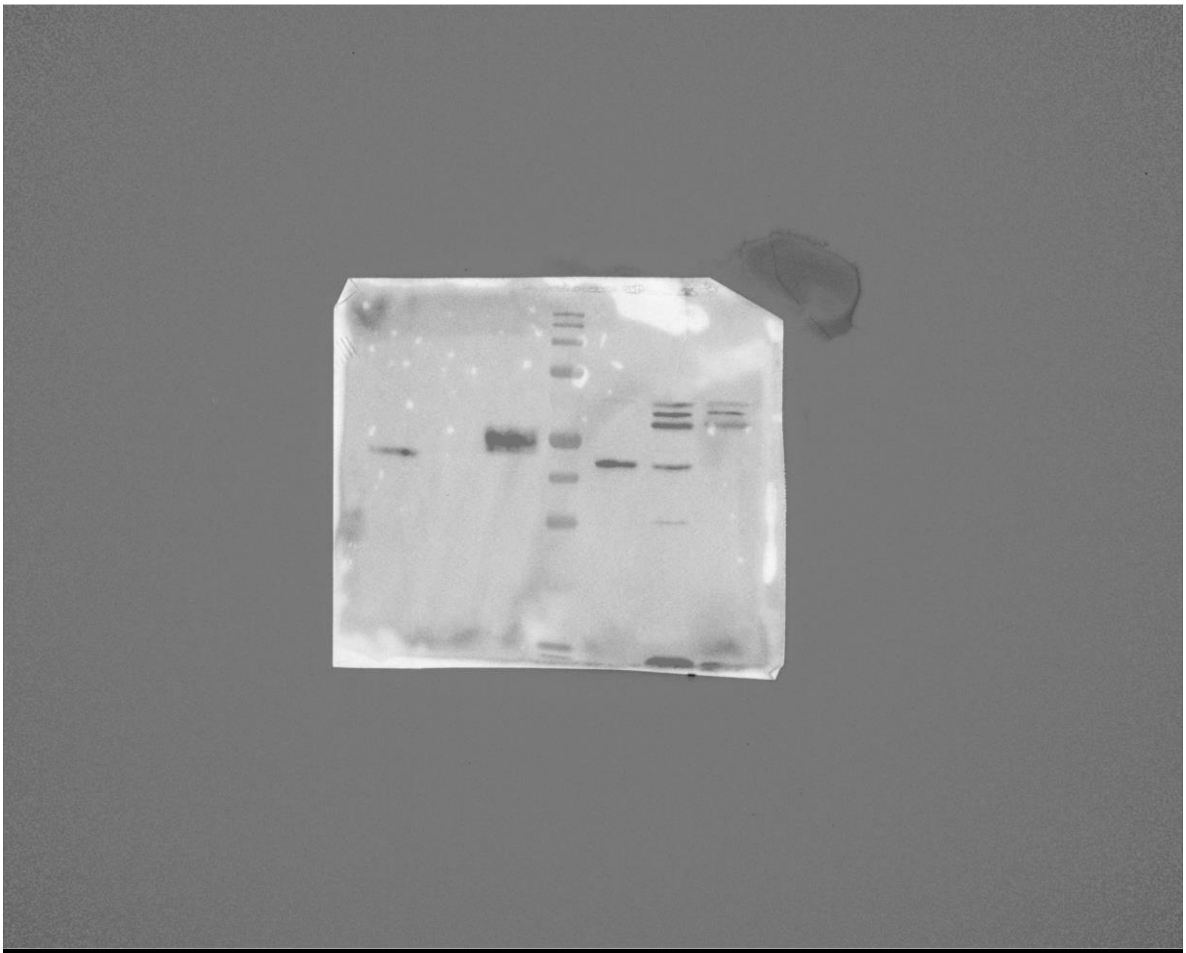
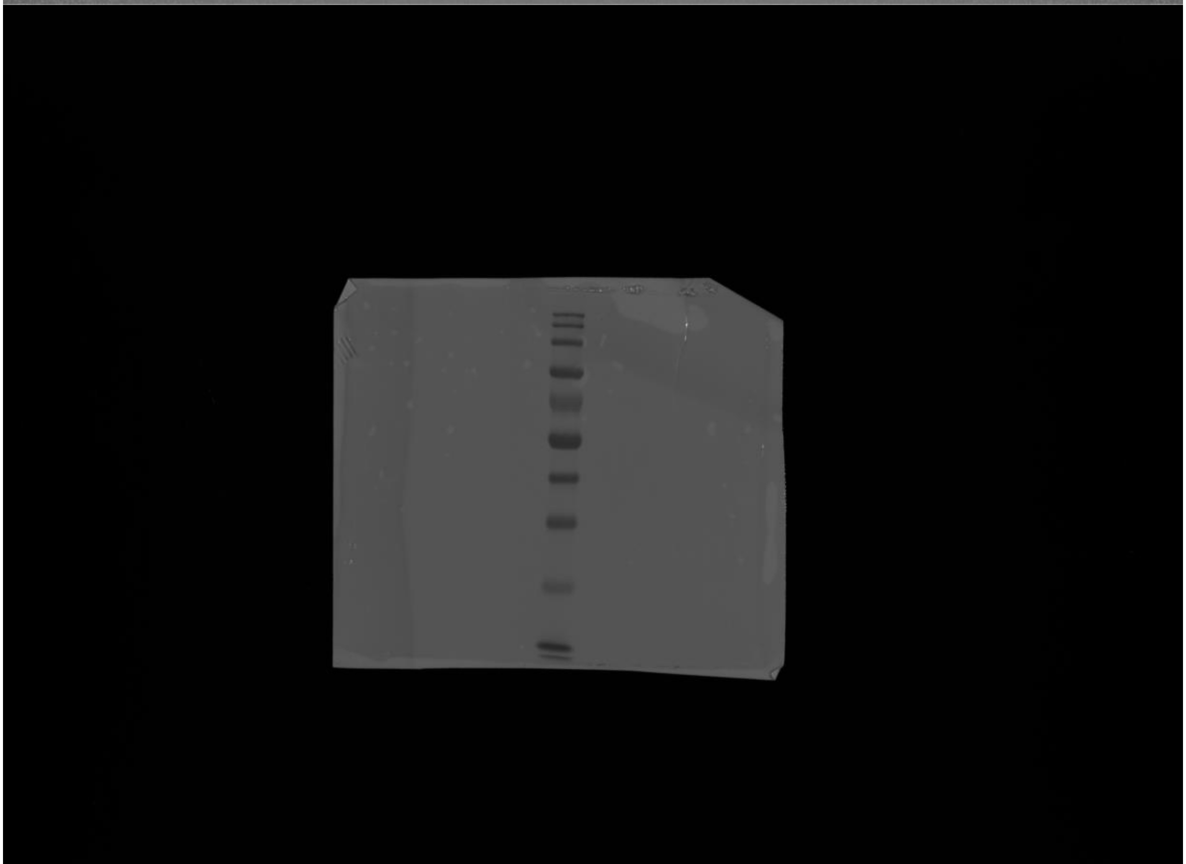

Supplement: Supplementary file 1 [file biology-15-01758-s001.zip › file s1-original images.pdf]
